# Resistant Starches Types 2 and 4 Have Differential Effects on the Composition of the Fecal Microbiota in Human Subjects

**DOI:** 10.1371/journal.pone.0015046

**Published:** 2010-11-29

**Authors:** Inés Martínez, Jaehyoung Kim, Patrick R. Duffy, Vicki L. Schlegel, Jens Walter

**Affiliations:** Department of Food Science and Technology, University of Nebraska, Lincoln, Nebraska, United States of America; Charité Campus Benjamin Franklin, Germany

## Abstract

**Background:**

To systematically develop dietary strategies based on resistant starch (RS) that modulate the human gut microbiome, detailed *in vivo* studies that evaluate the effects of different forms of RS on the community structure and population dynamics of the gut microbiota are necessary. The aim of the present study was to gain a community wide perspective of the effects of RS types 2 (RS2) and 4 (RS4) on the fecal microbiota in human individuals.

**Methods and Findings:**

Ten human subjects consumed crackers for three weeks each containing either RS2, RS4, or native starch in a double-blind, crossover design. Multiplex sequencing of 16S rRNA tags revealed that both types of RS induced several significant compositional alterations in the fecal microbial populations, with differential effects on community structure. RS4 but not RS2 induced phylum-level changes, significantly increasing Actinobacteria and Bacteroidetes while decreasing Firmicutes. At the species level, the changes evoked by RS4 were increases in *Bifidobacterium adolescentis* and *Parabacteroides distasonis*, while RS2 significantly raised the proportions of *Ruminococcus bromii* and *Eubacterium rectale* when compared to RS4. The population shifts caused by RS4 were numerically substantial for several taxa, leading for example, to a ten-fold increase in bifidobacteria in three of the subjects, enriching them to 18–30% of the fecal microbial community. The responses to RS and their magnitudes varied between individuals, and they were reversible and tightly associated with the consumption of RS.

**Conclusion:**

Our results demonstrate that RS2 and RS4 show functional differences in their effect on human fecal microbiota composition, indicating that the chemical structure of RS determines its accessibility by groups of colonic bacteria. The findings imply that specific bacterial populations could be selectively targeted by well designed functional carbohydrates, but the inter-subject variations in the response to RS indicates that such strategies might benefit from more personalized approaches.

## Introduction

The gastrointestinal microbiota is of profound importance for the human host, affecting its metabolism, immune functions, and physiology with implications to health [1], [2]. Not only are these microbial populations involved in the prevention of gastrointestinal infections and stimulation of the immune system, but recent research has indicated a role of the gut microbiome in complex diseases such as colon cancer, obesity, type 2 diabetes, and inflammatory bowel disease [3], [4], [5]. The implications of these cohesions cannot be overstated; if the gut microbiota influences health, it stands to reason that dietary factors which influence species composition and metabolic characteristics of the gut microbiota are strong candidates for disease prevention and intervention. Dietary components that are resistant to human digestion are considered the most significant source of nutrients for colonic bacteria, and they thus offer a promising tool for the modulation of the gut microbiota [6].

Resistant starches (RS) are starches or products of starch degradation that escape digestion and are not absorbed in the small intestine of healthy individuals [7]. RS are classified into four categories according to the features that render it undigestible. RS type 1 is physically inaccessible starch whereas RS type 2 (RS2) is native granular starch consisting of ungelatinized granules. RS type 3 is retrograded amylose, and finally, RS type 4 (RS4) is chemically modified to achieve undigestibility. Several studies have shown RS have the potential to improve health, with one of the primary benefits being maintenance of healthy blood sugar levels [8], [9]. Though resistant to digestion in the small intestine, bacterial species that reside in the colon are capable of utilizing RS as a substrate. These fermentations lead to an increase of short chain fatty acids (SCFA), especially butyrate, and a reduction of secondary bile acids, phenol, and ammonia [6], [10]. These metabolic effects are likely to underlie some of the documented health benefits of RS, which include the prevention of colon cancer development and colitis in animal models [11], [12], [13], [14].

Several studies have been performed to characterize the potential of RS to induce alterations in the composition of the gut microbiota. Increases in bifidobacteria [15], [16], [17], [18] and *Bacteroides* [19] as well as decreases in enterobacteria and *Bacteroides* [20] have been reported. Unfortunately, most studies have been performed in either *in vitro* systems or animal models [21]. To our knowledge, there were two previous studies that have used culture independent methods to characterize the effect of RS in humans *in vivo*. Abell and coworkers, who used denaturing gradient gel electrophoresis (DGGE) to study the impact of RS2 on the human gut microbiota and revealed an enrichment of phylotypes related to *Ruminococcus bromii* [22]. Walker et al (2010) detected an enrichment of bacteria related to *Eubacterium rectale* and *Ruminococcus bromii* when RS3 was consumed by overweight individuals [23].

Recently, the ecological study of the human gastrointestinal microbiota has gained enormous momentum through the development of high throughput multiplex sequencing of 16S rRNA tags [24], [25]. This technique has been extremely valuable, for example, in the characterization of the human microbiota in terms of lean and obese physiological states, impact of antibiotics, and the importance of delivery mode at birth [26], [27], [28]. Pyrosequencing has significant advantages over other molecular techniques currently used to study microbial communities. First, unlike probe based techniques such as fluorescence in-situ hybridization (FISH), pyrosequencing allows the determination of the entire phylogenetic spectrum of the bacterial populations in one single analysis. Second, it further allows an immediate taxonomic characterization and the flexibility to analyze the communities at different taxonomic levels. Third, pyrosequencing has a markedly increased dynamic range when compared to more traditional fingerprinting techniques such as DGGE [25].

**Figure 1:**
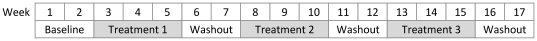
Experimental design used in this study. Subjects (n = 10) participated in a 17-week double-blind crossover design, in which 3 dietary treatments were assessed: 100 g of crackers containing either native starch or 33 g of RS2 or RS4. An initial baseline period was proceeded by 3-week periods of each dietary treatment in succession interspersed by 2-week washout periods, and a final washout period. Weekly fecal samples were collected throughout the entire study.

The goal of the present study was to obtain a community wide perspective of the impact of RS on the composition of the human gut microbiota. We were further interested to compare RS4 and RS2 in this respect because most emphasis in the literature has been placed on the latter substrate. To achieve our goal, we conducted a placebo-controlled, double-blind crossover trial with 10 human subjects and performed a comprehensive characterization of their fecal microbiota by using a combination of approaches, including pyrosequencing of 16S rRNA tags (Figure 1).

## Results

### Multiplex sequencing of 16S rRNA tags revealed alterations of the fecal microbiota through RS consumption and functional differences between RS types 2 and 4

Pyrosequencing of 16S rRNA amplicons from 161 fecal samples resulted in an average of 3,423 sequences per sample after quality control (551,183 sequences in total) with a mean sequence length of approximately 490 bp. The average number of operational taxonomic units (OTUs) identified per subject was 1,081. Rarefaction curves for all ten subjects and the three treatments and baselines/washouts were generated and are shown Figure S1A. This analysis and diversity examination by Shannon's index revealed that the consumption of RS did not alter the bacterial diversity in fecal samples (Figure S1B).

The bacterial composition in the ten subjects during the baseline period was, as shown by other studies [28], [29], dominated by the phyla Firmicutes (78%) and Bacteroidetes (13%). Other phyla detected were Actinobacteria (3%), Verrucomicrobia (1%), and Proteobacteria (1%), and 4% of the sequences remained unclassified (Figure S2A). At the family level, the predominant groups were the Lachnospiraceae (42%), Ruminococcaceae (19%), Bacteroidaceae (8%) (Figure S2B). Among the well characterized culturable genera were *Bacteroides* (7.5%), *Bifidobacterium* (1.3%), *Fecalibacterium* (8.4%), *Ruminococcus* (2.5%), *Roseburia* (2.1%), and *Dorea* (3.2%) (Figure S2C).

Sequence proportions determined by pyroseqeuncing were used to establish the effects of RS on the gut microbiota composition, and the groups of colonic bacteria that were significantly affected are shown in Table 1. The control crackers included in the study (providing a daily dose of more than 55 gram of native starch) did not have a significant impact on the fecal microbiota, as the microbial populations during administration of these crackers showed little difference to those during baseline and washout periods. In contrast, RS significantly affected several groups of colonic bacteria, with the two types of RS exerting functional differences in terms of their ability to modulate the gut microbiota. Taxonomy-based analysis using RDP Classifier revealed major differences in the proportions of phyla associated with consumption of RS4, including significant decreases in Firmicutes (*p*<0.001) by more than 10% on average, and increases in Bacteroidetes (*p*<0.01) and Actinobacteria (*p*<0.05) by around 5% each (Figure S2A). These changes were associated with a decrease in the family Ruminococcaceae (*p*<0.01) and increases in the genera *Parabacteroides* (*p*<0.001) and *Bifidobacterium* (*p*<0.05). The proportion of the genus *Faecalibacterium* decreased in the RS4 treatment (*p*<0.05), although this reduction was small (less than 1% when compared to the baseline) (Table 1). The genus *Dorea* was determined to be significantly reduced by both types of RS (p<0.01).

**Table 1:** Abundance of bacterial taxa that were impacted by RS consumption in fecal samples of ten human subjects as determined by pyrosequencing of 16S rRNA tags.

Proportion of bacterial taxa expressed in percentage (Mean ± SD)						
	RS2^1^	RS4^1^	Control^1^	Baseline^2^	Washout^3^	P-value^4^
Phylum						
Firmicutes	**75.9±13.4**	*65.6±15.0*	**79.6±9.6**	78.2±7.5	78.1±8.5	0.0010
Bacteroidetes	*10.1±6.6*	**16.3±9.7**	*10.4±6.9*	12.7±6.5	12.2±5.8	0.0028
Actinobacteria	6.1±6.4	**11.4±12.5**	*4.1±3.1*	3.1±2.5	4.1±3.2	0.0334
Family						
Bifidobacteriaceae	5.8±6.0	**11.1±11.7**	*3.0±2.5*	2.1±1.7	2.8±2.2	0.0262
Porphyromonadaceae	*0.6±1.0*	**3.4±1.9**	*0.5±0.3*	0.6±0.4	0.5±0.4	0.0002
Ruminococcaceae	**24.8±13.6**	*16.7±7.4*	**23.2±9.7**	19.3±7.4	20.7±7.6	0.0031
Erysipelotrichaceae	3.1±2.8	*2.6±2.6*	**3.9±3.2**	4.7±4.9	3.9±3.1	0.0279
Genus						
*Faecalibacterium*	9.7±4.4	*7.8±3.4*	**10.8±4.7**	8.4±4.2	8.8±2.9	0.0336
*Parabacteroides*	*0.6±1.0*	**3.4±1.9**	*0.4±0.5*	0.5±0.3	0.5±0.4	0.0002
*Bifidobacterium*	4.5±4.9	**8.9±10.2**	*2.2±1.7*	1.5±1.3	2.1±1.6	0.0342
*Dorea*	*1.7±1.2*	*1.6±1.2*	**3.0±2.0**	2.9±2.2	2.7±2.0	0.0030
Species (OTUs)						
*B. adolescentis*	3.7±4.5	**7.9±10.3**	*1.7±1.9*	1.5±1.2	1.8±1.3	0.0347
*P. distasonis*	*0.2±0.4*	**1.5±1.0**	*0.2±0.1*	0.2±0.1	0.2±0.2	0.0002
*R. bromii*	**4.1±5.1**	*1.2±1.3*	2.6±3.2	1.0±1.1	2.0±1.5	0.0479
*F. prausnitzii*	4.8±2.6	*3.6±2.0*	**5.6±3.1**	4.2±2.8	4.2±2.4	0.0160
*E. rectale*	**8.3±7.1**	*3.4±2.3*	4.9±4.0	5.4±2.9	4.7±2.0	0.0301
*D. formicigenerans*	1.2±1.0	*1.0±1.1*	**2.2±1.6**	2.3±1.8	1.9±1.7	0.0140
*C. clostridioforme*	2.6±2.4	**3.4±2.5**	*1.2±0.8*	1.4±1.3	1.5±1.2	0.0126
Clostridiales spp.	*0.3±0.6*	**0.9±0.9**	0.7±0.8	0.2±0.4	0.8±0.7	0.0322

1 The bacteria populations are averages of all three time points of feeding periods.

2 The bacteria populations are averages of the two time points of the baseline period.

3 The bacteria populations are averages of all the six time points of the three washout periods.

4 Bacterial populations during the dietary treatments were compared to each other with repeated measures ANOVA and Tukey's post hoc test. Numbers in bold represent proportions that were significantly higher than numbers shown in italic.

**Figure 2:**
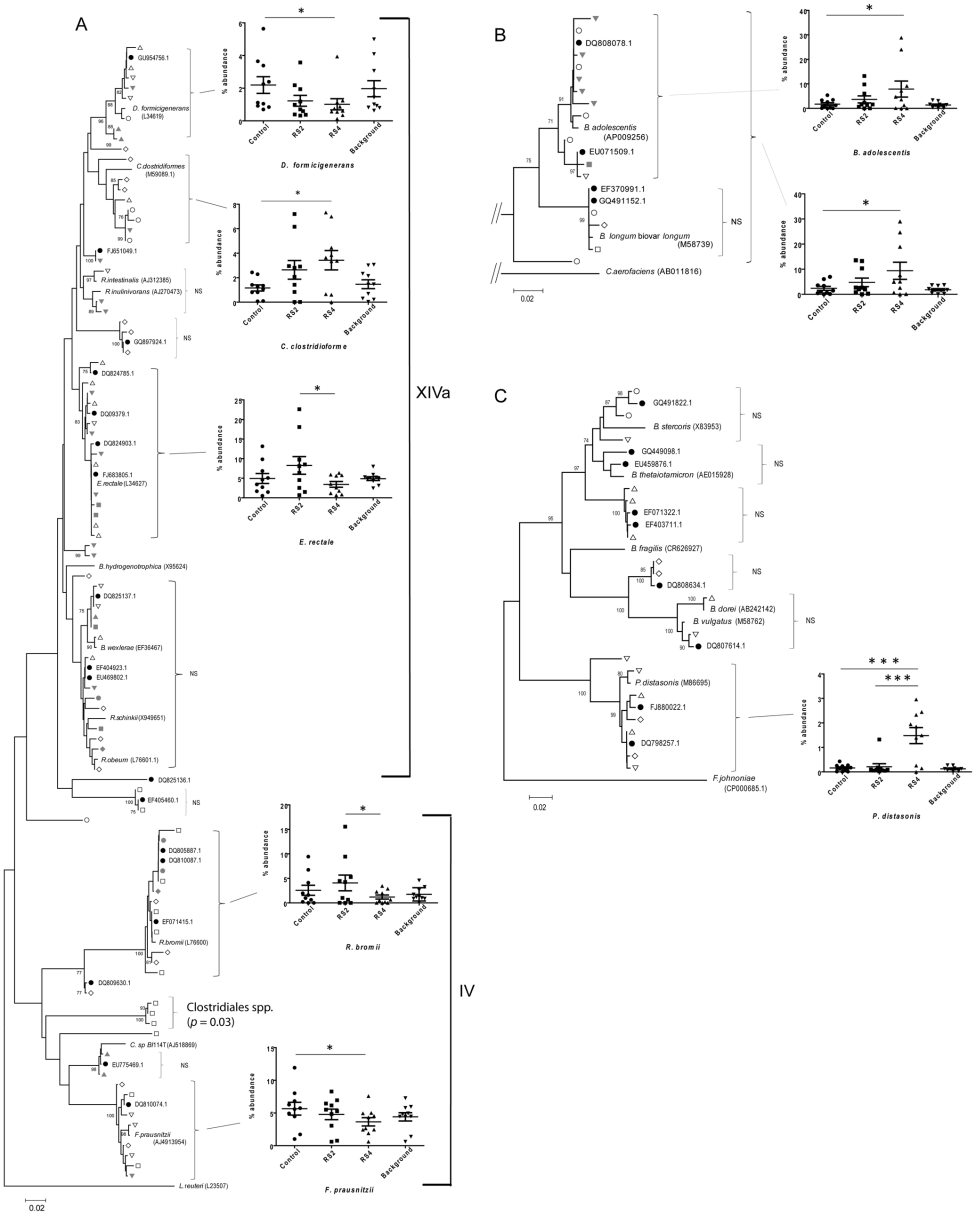
Characterization of the fecal microbiota in ten human subjects that consumed a random succession of crackers containing RS2, RS4, and native wheat starch (control) by multiplex pyrosequencing of 16S rRNA tags. Phylogenetic trees that encompass the phyla (A) Firmicutes (with Clostridiales groups XIVa and IV labeled), (B) Actinobacteria and (C) Bacteroidetes are shown. The trees contain representative sequences of all OTUs detected to be impacted by RS in individual subjects together with sequences of related entries in the database (which included both type strains of known species and sequences from molecular studies of human fecal samples). Sequences were aligned in Muscle 3.6 and the trees were built using the neighbor-joining algorithm with 1,000 bootstrap replicates in MEGA 4.0. Open-black and closed-gray symbols were used to label sequences from individual subjects. OTUs that were not significantly affected in all ten subjects were labeled as ‘not significant’ (NS). The graphs next to the trees show the abundance of OTUs and bacterial groups that were significantly altered in the treatment groups (RS2, RS4, control). These graphs show mean proportions of the three individual samples taken during the treatment periods for each subject. Background refers to samples taken in periods were no crackers were consumed. Repeated measures ANOVA in combination with a Tukey's post-hoc test were performed to indentify differences between treatment groups, and the background was not included in the statistic analysis. *p<0.05; **p<0.01; ***p<0.001.

To gain a more in depth understanding of the effects of RS on the relative abundances of microbial taxa, we used a phylogeny-based strategy to analyze the sequence data on the basis of OTUs (>97% sequence identity). First we identified OTUs that were affected through the dietary treatments in individual subjects. We then constructed phylogenetic trees with representative sequences of these OTUs according to phylum, which are shown in Figures 2A (for Firmicutes), 2B (for Actionobacteria), and 2C (Bacteroidetes). The abundance of the OTUs in all ten subjects was then quantified by local BLASTn. This analysis revealed that eight OTUs showed statistically significant differences among treatment groups, seven of which could be linked to known bacterial species (Table 1, Figure 2). The findings of the OTU-based approach were in general agreement with those obtained with the Classifier tool, and the species responsible for the significant RS induced changes in the genera *Bifidobacterium*, *Parabacteroides*, *Faecalibacterium*, and *Dorea* did correspond to *Bifidobacterium adolescentis* (*p*<0.05), *Parabacteroides distasonis* (*p*<0.001), *Faecalibacterium prausnitzii* (*p*<0.05) and *Dorea formicigenerans* (*p*<0.05), respectively (Table 1). In addition, the OTU-based analysis identified four additional taxa that differed between the treatment groups, belonging to the *Clostridium* clusters XIVa and IV. The proportion of *Clostridium clostridioforme* was increased by both RS, and the increase reached statistical significance for RS4 (*p*<0.05). Furthermore, the abundance of the species *Eubacterium rectale* (*p*<0.05) and *Rumminococcus bromii* (*p*<0.05) were significantly increased when RS2 was consumed when compared to RS4.

### The population shifts induced by RS were substantial but varied between subjects

**Figure 3:**
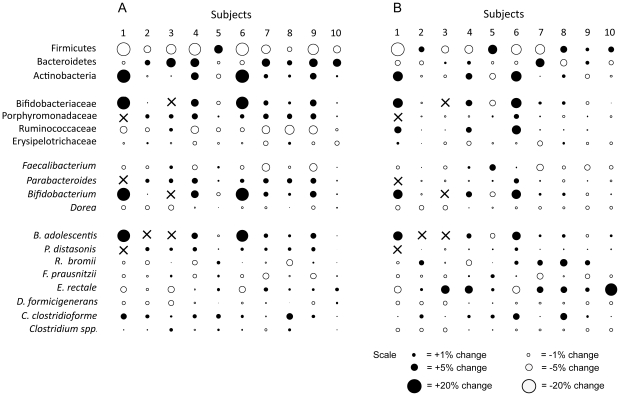
Bubble plots showing differences in the proportions of bacterial taxa (as per cent of the total microbiota composition) detected between the RS4 (A) and RS2 (B) periods when compared to the control period. The sizes of the bubbles are proportional to the magnitude of the difference. Black circles represent increases in proportions induced through RS treatment, and white circles show a decrease.

RS, and especially RS4, led to major changes in the composition of the gut microbiota in a subset of subjects. Numerically, the most substantial alterations were the change in the genus *Bifidobacterium* (e.g. *Bifidobacterium adolescentis*), which increased approximately 10 fold (from 2–3% to 18–30%) in three subjects through RS4. Other significant changes were *Parabacteroides distasonis*, which significantly increased through RS4 by 7 fold on average, and *Eubacterium rectale*, which was significantly enriched when RS2 was consumed, reaching around 20% of the total population in two of the subjects. Despite these substantial population shifts, our findings clearly showed that effects of RS and their magnitude varied among individuals. Figure 3 shows compositional changes induced by RS2 and RS4 when compared to administration of native starch for individual subjects. The data revealed that none of the community shifts induced through the two RS types were observed in all ten subjects. The most consistent alteration detected was the reduction in Firmicutes by RS4, which occurred in nine of the subjects (Figure 3). Other common alterations were the increase in Bacteroidetes (seven subjects), *Parabacteroides distasonis* (seven subjects), and *Bifidobacterium adolescentis* (six subjects) through RS4, and the increase of *Eubacterium rectale* through RS2 (eight subjects).

### Temporal dynamics of microbial populations in response to RS

**Figure 4:**
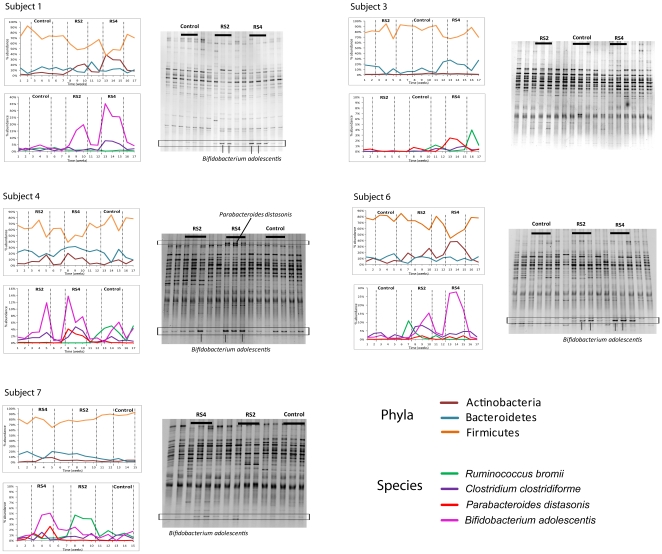
Temporal dynamics of the human fecal microbiota in response to the consumption of crackers containing RS2, RS4, and native wheat starch (control) in five human subjects. Graphs on the left show proportions of the three main phyla and four representative species (*Bifidobacterium adolescentis, Parabacteroides distasonis, Ruminococcus bromii* and *Clostridium clostridioforme*) as determined by pyrosequencing of 16S rRNA tags. Gel images on the right show molecular fingerprints generated by DGGE. Bands that represent *Bifidobacterium adolescentis* and *Parabacteroides distasonis* are labeled.

The generation of community profiles from 17 individual samples per subject throughout the trial allowed insight into how RS influenced the population dynamics within the fecal microbiota. This analysis showed that all the changes induced by RS were reversible within one week, and no differences in the proportions of the bacterial groups were detected between the first washout sample and the baseline (Student's *t*-test, *p*>0.05). Figure 4 shows the temporal patterns of the three main phyla (Actinobacteria, Bacteroidetes, and Firmicutes) and four selected species (*Ruminococcus bromii*, *Clostridium clostridioforme*, *Parabacteroides distasonis* and *Bifidobacterium adolescentis*) for five representative subjects. The data revealed that bacterial groups showed marked differences in the stability of their populations and in their temporal response to RS. For example, levels of *Bifidobacterium adolescentis* and *Parabacteroides distasonis* were remarkably stable in fecal samples in baseline and washout samples, and their populations returned to baseline level within one week after RS administration was stopped. In contrast, proportions of some taxa, e.g. the species *Ruminococcus bromii* and *Clostridium clostridioforme* showed higher fluctuations in background samples (Figure 4). Although all these taxa were also significantly impacted by dietary RS, population dynamics were more idiosyncratic, and these bacterial groups might be more influenced by other dietary components or environmental factors.

The analysis of population dynamics revealed that RS2 and RS4 induced changes within the fecal microbial community that differed in their temporal patterns. RS4 led to an abrupt increase in the abundance of *Bifidobacterium adolescentis* in some subjects with a slight mitigation throughout the three-week feeding period (subject 1, 4, and 6). Out of the six subjects that showed an increase in *Bifidobacterium adolescentis* with RS4, five did also manifest an increase with RS2. However, RS2 induced a slower gradual increase with higher proportions in week three of consumption when compared to week one.

### Selective culture, PCR-DGGE and *Bifidobacterium* specific quantitative RT-PCR (qRT-PCR)

Quantification of bacterial taxa in human fecal samples by pyrosequencing of 16S rRNA tags has been validated and it showed a high correlation with other molecular methods such as qRT-PCR and phylogenetic microarray [30], [31]. To investigate the impact of RS on human fecal microbiota with independent and well established methods, we analyzed all 161 fecal samples obtained during this study with selective culture for representative bacterial groups, PCR-DGGE, and *Bifidobacterium* specific qRT-PCR.

Throughout the study, numbers of total anaerobic bacteria, enterobacteria, enterococci, bifidobacteria, and *Bacteroides*/*Parabacteroides* spp. were determined by selective culture. These bacterial taxa were selected as they were included in previous studies that concerned the effect pre- and probiotics on the human fecal microbiota, and enterococci and enterobacteria cannot be detected by pyrosequencing as they constitute a minor fraction of the total microbial community in the human gut [32], [33]. Selective culture revealed significant higher numbers of bifidobacteria and *Bacteroides*/*Parabacteroides* spp. during RS4 consumption (*p*<0.05) (Table S1), confirming the findings obtained by pyrosequencing (Table 1). No significant changes were detected for enterococci and lactose fermenting enterobacteria (Table S1).

DGGE revealed that RS4, and to a lesser degree RS2, induced significant alterations to the fecal microbiota of most subjects. The most consistent change detected was a significant increase in the staining intensity of a DNA fragment that represented *Bifidobacterium adolescentis*, which occurred in four subjects during RS4 consumption and in 3 subjects when RS2 was consumed. The intensity of this DGGE band in all the subjects showed a remarkably high correlation (*r* = 0.9178, *p*<0.0001) with the abundance of *Bifidobacterium adolescentis* assessed by pyrosequencing (Figure S3A). DGGE analysis confirmed the distinct dynamics of the *Bifidobacterium adolescentis* population in response to RS2 and RS4 that were detected by pyrosequencing, meaning that RS4 induced a swift and reversible increase in band intensity while RS2 caused a gradual raise (Figure 4). DGGE also detected an increase of *Parabacteroides distasonis* in one of the subjects when RS4 was consumed. Therefore, DGGE confirmed the increase in bifidobacteria and *Parabacteroides distaso*nis in some of the subjects, but as expected, overall resolution of this technique was lower than pyrosequencing.

Quantitative enumeration of bifidobacteria by qRT-PCR confirmed the significant increase in *Bifidobacterium* numbers during consumption of RS4 (10.3±1.5 log10 cells/gram of feces; *p*<0.01), and also indicated a significant increase when RS2 (10.1±1.3 log10 cells/gram of feces; *p*<0.05) was consumed compared to control (9.7±1.2 log10 cells/gram of feces). The total cell numbers of bifidobacteria increased by more than three-fold on average through RS4, while RS2 doubled the numbers. In the three subjects with the highest response to RS4, qRT-PCR showed an increase of bifidobacteria to more than 10^11^ cells per gram. The cell counts obtained with qRT-PCR in all fecal samples included in this study (n = 161) showed a high correlation (*r* = 0.8310, *p*<0.0001) with the abundance of bifidobacteria determined by pyrosequencing (Figure S3B).

### RS was well tolerated by human subjects

In addition to microbiota analyses, we also collected data on bowel-related characteristics during the feeding and washout periods using a weekly symptoms diary that rated bowel movement, stool consistency, discomfort, flatulence, abdominal pain, and bloating on a scale from 1 (best) to 5 (worse). One-way ANOVA analysis revealed a significant difference for flatulence (*p*<0.05), which was moderately increased during the consumption of both types of RS when compared to periods with control crackers (Table S2). No significant changes occurred in fecal pH for any of the treatments and no significant detrimental effects were observed on bowel movement, stool consistency, or discomfort, indicating that RS at doses of 33 g per day are well tolerated in human subjects.

## Discussion

To gain a deeper understanding of the impact of two chemically different forms of RS on the composition and temporal dynamics of the fecal microbiota, we employed a study in 10 human subjects who were weekly monitored throughout a period of 17 weeks. The data revealed that RS types induced substrate specific shifts in the fecal microbial community that were tightly associated with consumption and which varied between subjects. Our *in vivo* findings on RS2 were in accordance to previous studies on starch fermentation in *in vitro* models of the large intestine, which showed an enrichment of *Bifidobacterium adolescentis*, *Eubacterium rectale*, and *Ruminococcus bromii* [34], [35]. The same bacterial groups, with the exception of bifidobacteria, were also enriched in fecal samples of obese human subjects during consumption of RS3 [23]. In contrast, our findings clearly showed that the response of the fecal microbiota to RS4 differed to that of RS2 and RS3. For example, *Parabacteroides distasonis* was enriched through RS4, while *Eubacterium rectale* and *Ruminococcus bromii* showed a significant decrease. Strikingly, RS4 also led to phylum level alterations, decreasing the proportion of Firmicutes while increasing Bacteroidetes and Actinobacteria. Such phylum level changes have not been observed in fecal samples of human subjects consuming RS2 in our study and RS3 as shown by Walker and coworkers [23].

There was very little overlap in the bacterial groups that responded to both RS2 and RS4. This was surprising as the RS types used in this study were both starch polysaccharides that consist of glucose monomers with the same covalent bonds, although the RS4 was cross-linked by phosphorylation. One of the bacterial groups that responded to both RS types was the genus *Bifidobacterium*, which increased in six subjects with RS4 and in five of the same subjects with RS2. However, the temporal dynamics of these modulations differed. RS2 led to a much slower raise in bifidobacteria, reaching comparable numbers to RS4 only in week three. This clearly showed that time is an important variable when studying dietary modulations of the human gut microbiota. It appears that the ability to increase levels of bifidobacteria is comparable between RS2 and RS4 in the long run, but extended feeding studies will be necessary to determine the exact taxon-time patterns of responses to different forms of RS.

Questions remain about the mechanisms by which different RS types selectively promote groups of colonic bacteria in humans *in vivo*. Starch fermentation per se should not be selective as many bacterial genera (*Clostridium*, *Bacteroides*, *Bifidobacterium, Butyrivibrio*, *Prevotella*, *Roseburia*, *Eubacterium*, *Ruminococcus*, etc.) present in the human GIT can utilize this substrate *in vitro*, and various bacterial systems involved in the degradation of starch have been identified by genomic approaches [6], [34], [36], [37], [38], [39], [40], [41], [42], [43]. However, our *in vivo* findings showed that substrate preferences and competitive abilities exist in the gut environment. In this respect, it is important to point out that the RS types used in this study varied markedly in terms of their chemical structure. RS2 is a granular form of high amylose corn starch, while RS4 is a chemically modified starch that is cross-linked through phosphate moieties. Therefore, it is possible that different groups of colonic bacteria produce enzymes with distinct activities towards the two RS types, promoting different dynamics in the gut ecosystem. However, it is also of note that the ability of bacterial groups to make use of RS *in vivo* might relate not only to the utilization but also to the binding of the substrate. It is striking that *Ruminococcus bromii*, *Bifidobacterium adolescentis*, and *Eubacterium rectale*, which showed the most substantial increases in the human gut in response to RS2, have also been shown to form highly selective associations with this substrate [35]. Therefore, the adherence of bacteria to starch granules might constitute an important first step in the utilization of this substrate, and groups of colonic bacteria might differ in their ability to adhere to granules of RS2 and RS4. The mechanisms by which different types of RS become fermented in the human colon remain an important area of future research.

A significant finding of this study was the individualized responses of the gut microbiota to RS2 and RS4, which has also been shown previously for RS3 [23]. Out of the nineteen OTUs that were detected to respond during this study in individual subjects, eleven did not reach significance when all subjects were included in the analysis (e.g. *Bifidobacterium longum*, *Ruminococcus obeum*, *Roseburia intestinalis*, *Roseburia inulinivorans*, and several *Bacteroides spp.*). In addition, none of the taxa that were significantly affected by RS showed a response in all ten subjects. There are three possible explanations for the individuality of the responses. First, few OTU are completely conserved among humans [28], [44], thus species that were affected in some subjects might simply not be present in other individuals. Second, strain-level differences in the ability to utilize substrates could contribute to the inter-individual variations. For example, it has been shown for *Bifidobacterium adolescentis* that individual strains can have major differences in their amylolytic activity [45], [46]. Lastly, host factors might play an important role. For example, subject specific environmental constrains (e.g. through limitations in growth factors other than carbohydrates) might restrict the ability of a bacterial group to increase in numbers even if a suitable growth substrate is provided. In addition, differences in host genotype might influence transit times or the amount of digestive enzymes secreted, thus affecting the fraction of RS that survives digestion.

There is currently no scientific consensus of what defines a healthy human microbiota in terms of its composition. Therefore, predictions on the consequences of the compositional alterations induced through RS2 and RS4 in terms of health remain speculative. Nevertheless, the distinct effects of RS2 and RS4 on the microbial community in the gut suggest that these substrates could have a different impact on the host. RS2 promoted *Eubacterium rectale*, a species associated with high butyrate production [4], a trait that could be especially beneficial in the prevention of inflammation and colon cancer [47], [48]. In contrast, RS4 reduced the amount of Firmicutes in favor of Bacteroidetes and bifidobacteria. Such a shift in the gut microbiota could be especially beneficial in the prevention or treatment of obesity and related metabolic disorders. A microbiome enriched in Firmicutes has been associated with an increased capacity for energy harvest and obesity [49], [50], and a reduction of this phylum could therefore reduce the amount of calories extracted from the diet. Furthermore, bifidobacteria have been linked to metabolic and immunological improvements related to type 2 diabetes [51]. As we gain a better understanding about the contributions of members of the gut microbiota to disease, knowledge as obtained during this study can aid in a more systematic selection of carbohydrates for intervention studies.

In this study, we demonstrated that RS2 and RS4 promote distinct compositional alterations within the human gut microbiota. These functional differences imply that specific bacterial populations can be selectively targeted by starches with different chemical structures. If future research will reveal causative associations between dysbiosis and disease, then selective dietary strategies that redress these imbalances have potential to improve health. However, the individualized responses observed during this study certainly pose a hurdle to developing universal dietary recommendations, and they imply that more personalized strategies that target the gut microbiome might enhance the success rate of such applications.

## Materials and Methods

The human trial of this study was approved by the Institutional Review Board of the University of Nebraska (IRB Approval Number: 2008038840EP), and written informed consent has been obtained from all subjects.

### Preparation of RS crackers

Three types of crackers, containing either RS2 (Hi-Maize 260, National Starch and Chemical Corp., Bridgewater, N.J., USA), RS4 (Fibersym® RW, MGP Ingredients, Atchison, Kansas, USA), or native wheat starch (Midsol 50, MGP Ingredients, Atchison, Kansas, USA), were prepared at the American Institute of Baking International (Manhattan, Kansas). Fibersym® RW is a chemically modified phosphorylated cross-linked type 4 RS prepared from wheat starch (RS4) [52]. The crackers containing RS were formulated to both contain 33 g of fiber in the form of RS per 100 g of crackers, calculated based on the proportion of total dietary fiber (true RS) in Hi-Maize 260 (60%, dry basis) and Fibersym® RW (85%, dry basis), using native wheat starch to account for the different RS contents of Hi-Maize 260 and Fibersym® RW. The formulations of all three types of crackers are shown in Table S3, and the baking conditions of dough are presented in Table S4. The amount of RS in the final products was confirmed using the AOAC 991.41 method, which measures total fiber (and the most reliable method to measure RS4). This analysis revealed that the amount of fiber per 100 g of crackers after processing was 4.53 g±1.4 g for the control crackers, 33.2 g±4.2 g for the RS2 crackers and 30.5 g±3.5 g for the RS4 crackers.

### Experimental design of human study

A double-blind, crossover study was performed starting with 13 healthy human subjects. None of the subjects had been on antibiotics or on a vegetarian diet within three months prior to the start of the study or throughout its duration. Three subjects stopped their participation for reasons unrelated to the study. Thus, the study was completed by ten subjects (five males and five females) between 23 and 38 years of age. The study was conducted over a 17-week period, beginning with a two-week baseline period (no crackers administered). The subjects then consumed 100 gram per day of the different crackers in sequence, each for three weeks, interspersed by 2-week washout periods. The study finished with a 2-week washout. Fecal samples were collected weekly, resulting in a total of 161 fecal samples for the entire study. For reasons unrelated to the study, we were unable to collect a total of 9 fecal samples distributed among five subjects. All missing samples corresponded to washout periods, and their omission did therefore not affect the statistical analysis.

Subjects completed a symptoms diary to assess the potential side effects of RS administration. The symptoms included were bowel movement, stool consistency, discomfort, flatulence, abdominal pain, and bloating, and subjects were asked to score them on a scale from 1 (none, normal, good well-being) to 5 (severe symptoms and discomfort).

### Collection of fecal samples and analysis by selective culture

Fresh fecal samples were processed within an hour of defecation. A ten-fold dilution of each sample in sterile phosphate buffered saline (PBS) (pH 7.0) was immediately frozen at −80°C for later DNA extraction (see below). Samples were further introduced into an anaerobic chamber (Bactron IV Anaerobic Chamber, Shel Lab, USA), and a 10-fold dilution series was made with pre-reduced sterile saline (0.9% NaCl). Aliquots were plated on Brain Heart Infusion agar (BD, USA) for the enumeration of total anaerobes (2 days), Rogosa SL (BD) for bifidobacteria (4 days), and Bacteroides Bile Esculin Agar (BD) for *Bacteroides*/*Parabacteroides* spp. (2 days), and the plates were incubated anaerobically at 37°C. Dilution series were plated aerobically on MacConkey agar (BD,) for enterobacteria (1 day); and Bile Esculin Azide Agar (Acumedia, USA) for enterococci (2 days); plates were incubated aerobically at 37°C. Fecal samples (2 gram) were homogenized with distilled water to obtain a slurry for pH measurements, which were performed using an Ag/AgCl pH meter (Accumet Basic AB15 pH meter, Fisher Scientific).

### DNA extraction from fecal samples

Fecal homogenates were thawed and transferred to sterile bead beating tubes (Biospec products, Bartlesville, OK, USA) containing 300 mg of zirconium beads. Cells were recovered by centrifugation (8,000×g for 5 min at room temperature) and suspended in ice-cold PBS to wash the cells. This step was repeated twice before cell pellets were suspended in 100 µl of lysis buffer (200 mM NaCl, 100 mM Tris, 20 mM EDTA, 20 mg/ml Lysozyme, pH 8.0) containing 20 mg/ml of Lysozyme (Sigma-Aldrich) and incubated at 37°C for half an hour. Buffer ASL (1.6 ml) from QIAamp DNA Stool Mini Kit (Qiagen, Hilden, Germany) was added to each sample, after which the samples were homogenized in a MiniBeadbeater-8 (BioSpec Products, OK, USA) for 2 min at maximum speed. 1.2 ml of supernatant was used to purify DNA with the QIAamp DNA Stool Mini Kit following the manufacturer's instructions.

### Pyrosequencing of 16S rRNA tags

The V1-V3 region of the 16S rRNA gene was amplified by PCR from fecal DNA. The 16S primers were modified to work with the Roche-454 Titanium adapter sequences and contain the A or B Titanium sequencing adapter (shown in italics), followed immediately by a unique 8-base barcode sequence (BBBBBBBB) and finally the 5′ end of primer. A mixture (4∶1) of the primers B-8FM (5′- *CCTATCCCCTGTGTGCCTTGGCAGTCTCAG*AGAGTTTGATCMTGGCTCAG—3′) and B-8FMBifido (5′-*CCTATCCCCTGTGTGCCTTGGCAGTCTCAG* *AGGGTTCGATTCTGGCTCAG—3′* ) were used as the forward primer during PCR. As the reverse primer, the primer A-518R (5′- *CCATCTCATCCCTGCGTGTCTCCGACTCAG*BBBBBBBBATTACCGCGGCTGCTGG —3′) was used. Individual samples were amplified with primers containing unique barcodes, which allowed the mixing of PCR products from multiple samples in a single run, followed by bioinformatic assignation of the sequences to their respective samples via the barcode. Primer 8FMBifido was used in combination with primer 8FM to detect bifidobacteria, as species within this genus do not match the latter primer [31]. The PCR mixture contained 1 µl of forward primer mix, 1 µl of reverse primer, 0.25 µl of Ex-Taq polymerase (TaKaRa Bio, USA), 1.5 µl of the sample, 6.25 µl of Ex-Taq buffer, 5 µl of deoxynucleotides and 37 µl of sterile dH_2_O were used for the reaction. The PCR program consisted of an initial denaturing step for 5 min at 95°C, followed by 30 cycles of denaturation at 95°C for 45 sec, annealing at 57°C for 45 sec and extension at 72°C for 2 min, with a final step at 72°C for 10 min. The PCR products were quantified based on their staining intensity using the image acquisition software Genesnap (Syngene USA). PCR products were combined in equal amounts and gel purified using the QIAquick Gel Extraction Kit (Qiagen, USA).

Pyrosequencing was performed by the Core for Applied Genomics and Ecology (CAGE, University of Nebraska) from the A end with the 454/Roche A sequencing primer kit using a Roche Genome Sequencer GS-FLX following manufacturer's protocol for the Roche 454 GS FLX Titanium. Sequences were binned according to barcode using the ‘Initial Process’ tool of the Ribosomal Database Project (RDP) Pyrosequencing Pipeline (http://pyro.cme.msu.edu/) [53] with default parameters (which included the removal of sequences containing at least one ambiguous nucleotide), except for the minimum sequence length, which was set to 300 bp. The quality approved sequences were trimmed to 495 bp before their submission to the sequence analyses (see below).

### Sequence analyses to characterize microbial populations

Two independent approaches were used to analyze the sequences obtained with pyrosequencing. First, the Classifier tool (with a minimum bootstrap value of 80%) of the RDP was applied to obtain a taxonomic assignment of all sequences. This approach allowed a fast determination of the proportions of bacterial groups at different taxonomic levels (phylum, family, genus). Second, sequences were assigned to Operational Taxonomic Units (OTUs) that were quantified in individual subjects. As the entire data from the ten subjects contained too many sequences for a quality alignment, sequences were aligned by subject using the Aligner web tool of the RDP, and then clustered using the Complete Linkage Clustering tool (with a maximum distance cutoff of 97%). OTUs that contained less than three sequences were excluded from the analyses. ANOVA was used to identify OTUs that were significantly affected by the dietary treatments in each of the ten subjects. These OTUs were subjected to a taxonomic classification and grouped according to phylum (Firmicutes, Bacteroidetes, and Actinobacteria). Within these phyla, five random sequences of each OTU identified above were aligned with the most closely related type strains and entry in the NCBI database using Muscle 3.6 [54]. Phylogenetic trees were built with MEGA 4.0 Software [55] by neighbor-joining with 1,000 bootstrap replicates. These trees allowed us to visually assign OTUs as sequence clusters which, in most cases, encompassed sequences from several subjects, and consensus sequences were generated for each OTU. A local nucleotide database was established for each subject in BioEdit [56] containing all sequences detected by pyrosequencing, and the BLASTn algorithm was used with a 97% cutoff (min. length 300 bp) to quantify each OTU in the fecal bacterial populations in each sample. We verified that this approach did not result individual sequences being assigned to different OTUs. In two occasions, two OTUs that were initially identified as distinct had very high sequence similarities, and were thus combined.

Diversity of the fecal microbiota was determined using 16S rRNA sequence data with two different methods, Shannon's index and the generation of rarefraction curves. The DNA sequences of each sample were individually aligned and clustered using Aligner and Complete Linkage Cluster tools of the RDP. Individual cluster files corresponding to each fecal sample were used to determine the Shannon's Index and construct Rarefraction curves.

### Analysis of fecal microbiota by PCR-DGGE

PCR-DGGE and quantitative analysis of molecular fingerprints was performed as described previously [31]. Briefly, PCR was performed using primers PRBA338fGC (5′CGCCCGCCGCGCGCGGCGGGCGGGGCGGGGGCACGGGGGGACTCCTACGGGAGGCAGCAG’3) and PRUN518r (5′-ATTACCGCGGCTGCTGG-3′) (Ovreas *et al.*, 1997). DGGE was done as described by Walter and co-workers [57] using a DCode universal mutation detection system (Bio-Rad, Hercules, USA), and DGGE profiles were analyzed using BioNumerics software Version 5.0 (Applied Maths). Band staining intensities were calculated as percent peak area in relation to the total peak area of the entire fingerprint. DNA fragments whose staining intensity changed according to dietary treatment were excised, purified as described by Walter and coworkers [58], and cloned using the TOPO® TA Cloning® Kit for Sequencing (pCR® 4 TOPO® Vector) (Invitrogen). Plasmids were isolated from transformants using the QIAprep Spin Minprep kit (Qiagen, Hilden, Germany), and inserts were sequenced by a commercial provider. Closest relatives of the partial 16S rRNA sequences were determined using the SeqMatch web tool provided through the Ribosomal Database Project (http://rdp.cme.msu.edu/seqmatch/seqmatch_intro.jsp).

### *Bifidobacterium* specific qRT-PCR

Quantitative real time PCR (qRT-PCR) was performed as described by Martínez *et al.* [31], using a Mastercycler Realplex2 (Eppendorf AG, Hamburg, Germany) and the *Bifidobacterium*-specific primers (F: 5′TCGCGTC(C/T)GGTGTGAAAG’3) and R: 5′CCACATCCAGC(A/G)TCCAC’3) [59]. Standard curves for absolute quantification of bifidobacteria in the fecal samples were prepared using overnight cultures (14 h) of *Bifidobacterium animalis* ATCC 25527^T^ and *Bifidobacterium infantis* ATCC 15697^T^.

### Statistical analysis

One-way ANOVA tests with repeats were performed to identify differences in fecal microbiota composition induced through the dietary treatments (RS2, RS4 and control) in all ten subjects. One-way ANOVA tests were performed to identify significant alterations of taxa in individual subjects. Samples obtained during the baseline and washout periods were not included into the statistical analysis. Post hoc pair-wise comparisons were done using Tukey's method. *P*-values <0.05 were considered significant unless otherwise stated.

## Supporting Information

Table S1 Enumeration of bacterial groups through culturing.
(DOC)

Table S2 Mean ± standard deviations of weekly symptoms reported by the subjects in a scale from 1 (best) to 5 (worse).
(DOC)

Table S3 Formulation of crackers per 100 grams.
(DOC)

Table S4 Baking conditions (°F) of crackers containing control starch, RS2, RS4.
(DOC)

Figure S1 **Diversity and species richness of the fecal microbiota in ten human subjects that consumed crackers containing native starch (red), RS2 (green), RS4 (purple), or no crackers (yellow).** (A) Rarefaction curves showing the amount of OTUs in all individual fecal samples taken from the ten subjects. (B) Shannon's Diversity Index for all subjects during treatments and baseline/washout.
(PDF)

Figure S2 **Collective fecal microbial composition including the major taxonomic groups at the (A) phylum, (B) family, and (C) genus levels averaged for 10 human subjects corresponding to the baseline, washouts, and periods in which crackers containing native starch (control), RS2 and RS4 were consumed.**
(PDF)

Figure S3 **Confirmation of findings obtained with pyrosequencing by analyzing the fecal microbiota with PCR-DGGE and *Bifidobacterium* specific qRT-PCR.** (A) Pearson correlation between the abundance of *Bifidobacterium adolescentis* as determined by band intensity in PCR-DGGE and pyrosequencing of 16S rRNA tags. (B) Pearson correlation between cell numbers and percent abundance of bifidobacteria as determined by qRT-PCR and pyrosequencing, respectively.
(PDF)

## References

[1] Gordon HA, Pesti L (1971). The gnotobiotic animal as a tool in the study of host microbial relationships.. Bacteriol Rev.

[2] Nicholson JK, Holmes E, Wilson ID (2005). Gut microorganisms, mammalian metabolism and personalized health care.. Nat Rev Microbiol.

[3] Cani PD, Neyrinck AM, Fava F, Knauf C, Burcelin RG (2007). Selective increases of bifidobacteria in gut microflora improve high-fat-diet-induced diabetes in mice through a mechanism associated with endotoxaemia.. Diabetologia.

[4] Flint HJ, Duncan SH, Scott KP, Louis P (2007). Interactions and competition within the microbial community of the human colon: links between diet and health.. Environ Microbiol.

[5] Tannock GW (2008). The search for disease-associated compositional shifts in bowel bacterial communities of humans.. Trends Microbiol.

[6] Louis P, Scott KP, Duncan SH, Flint HJ (2007). Understanding the effects of diet on bacterial metabolism in the large intestine.. J Appl Microbiol.

[7] Asp NG (1992). Resistant starch. Proceedings from the second plenary meeting of EURESTA: European FLAIR-Concerted Action No 11 on the physiological implications of the consumption of resistant starch in man.. Eur J Clin Nutr.

[8] Behall KM, Scholfield DJ, Hallfrisch JG, Liljeberg-Elmstahl HG (2006). Consumption of both resistant starch and beta-glucan improves postprandial plasma glucose and insulin in women.. Diabetes Care.

[9] Nilsson AC, Ostman EM, Holst JJ, Bjorck IM (2008). Including indigestible carbohydrates in the evening meal of healthy subjects improves glucose tolerance, lowers inflammatory markers, and increases satiety after a subsequent standardized breakfast.. J Nutr.

[10] Nugent AP (2005). Health properties of resistant starch.. Nutr Bull.

[11] Bauer-Marinovic M, Florian S, Muller-Schmehl K, Glatt H, Jacobasch G (2006). Dietary resistant starch type 3 prevents tumor induction by 1,2-dimethylhydrazine and alters proliferation, apoptosis and dedifferentiation in rat colon.. Carcinogenesis.

[12] Le Leu RK, Brown IL, Hu Y, Esterman A, Young GP (2007). Suppression of azoxymethane-induced colon cancer development in rats by dietary resistant starch.. Cancer Biol Ther.

[13] Moreau NM, Martin LJ, Toquet CS, Laboisse CL, Nguyen PG (2003). Restoration of the integrity of rat caeco-colonic mucosa by resistant starch, but not by fructo-oligosaccharides, in dextran sulfate sodium-induced experimental colitis.. Br J Nutr.

[14] Toden S, Bird AR, Topping DL, Conlon MA (2006). Resistant starch prevents colonic DNA damage induced by high dietary cooked red meat or casein in rats.. Cancer Biol Ther.

[15] Conlon MA, Bird AR (2009). Interactive and individual effects of dietary non-digestible carbohydrates and oils on DNA damage, SCFA and bacteria in the large bowel of rats.. Br J Nutr.

[16] Drzikova B, Dongowski G, Gebhardt E (2005). Dietary fibre-rich oat-based products affect serum lipids, microbiota, formation of short-chain fatty acids and steroids in rats.. Br J Nutr.

[17] Kleessen B, Stoof G, Proll J, Schmiedl D, Noack J (1997). Feeding resistant starch affects fecal and cecal microflora and short-chain fatty acids in rats.. J Anim Sci.

[18] Wang X, Brown IL, Khaled D, Mahoney MC, Evans AJ (2002). Manipulation of colonic bacteria and volatile fatty acid production by dietary high amylose maize (amylomaize) starch granules.. J Appl Microbiol.

[19] Lesmes U, Beards EJ, Gibson GR, Tuohy KM, Shimoni E (2008). Effects of resistant starch type III polymorphs on human colon microbiota and short chain fatty acids in human gut models.. J Agric Food Chem.

[20] Silvi S, Rumney CJ, Cresci A, Rowland IR (1999). Resistant starch modifies gut microflora and microbial metabolism in human flora-associated rats inoculated with faeces from Italian and UK donors.. J Appl Microbiol.

[21] Crittenden R, Playne MR, Ouwehand AC, Vaughan EE (2006). Modifying the human intestinal microbiota with prebiotics.. Gastrointestinal microbiology.

[22] Abell GC, Cooke CM, Bennett CN, Conlon MA, McOrist AL (2008). Phylotypes related to Ruminococcus bromii are abundant in the large bowel of humans and increase in response to a diet high in resistant starch.. FEMS Microbiol Ecol.

[23] Walker AW, Ince J, Duncan SH, Webster LM, Holtrop G Dominant and diet-responsive groups of bacteria within the human colonic microbiota.. ISME J Epub ahead of print.

[24] Robinson CJ, Bohannan BJ, Young VB From structure to function: the ecology of host-associated microbial communities.. Microbiol Mol Biol Rev.

[25] Hamady M, Knight R (2009). Microbial community profiling for human microbiome projects: Tools, techniques, and challenges.. Genome Res.

[26] Dethlefsen L, Huse S, Sogin ML, Relman DA (2008). The pervasive effects of an antibiotic on the human gut microbiota, as revealed by deep 16S rRNA sequencing.. PLoS Biol.

[27] Dominguez-Bello MG, Costello EK, Contreras M, Magris M, Hidalgo G Delivery mode shapes the acquisition and structure of the initial microbiota across multiple body habitats in newborns.. Proc Natl Acad Sci U S A.

[28] Turnbaugh PJ, Hamady M, Yatsunenko T, Cantarel BL, Duncan A (2009). A core gut microbiome in obese and lean twins.. Nature.

[29] Ley RE, Turnbaugh PJ, Klein S, Gordon JI (2006). Microbial ecology: human gut microbes associated with obesity.. Nature.

[30] Claesson MJ, O'Sullivan O, Wang Q, Nikkila J, Marchesi JR (2009). Comparative analysis of pyrosequencing and a phylogenetic microarray for exploring microbial community structures in the human distal intestine.. PLoS One.

[31] Martinez I, Wallace G, Zhang C, Legge R, Benson AK (2009). Diet-induced metabolic improvements in a hamster model of hypercholesterolemia are strongly linked to alterations of the gut microbiota.. Appl Environ Microbiol.

[32] Tannock GW, Munro K, Bibiloni R, Simon MA, Hargreaves P (2004). Impact of consumption of oligosaccharide-containing biscuits on the fecal microbiota of humans.. Appl Environ Microbiol.

[33] Tannock GW, Munro K, Harmsen HJ, Welling GW, Smart J (2000). Analysis of the fecal microflora of human subjects consuming a probiotic product containing Lactobacillus rhamnosus DR20.. Appl Environ Microbiol.

[34] Kovatcheva-Datchary P, Egert M, Maathuis A, Rajilic-Stojanovic M, de Graaf AA (2009). Linking phylogenetic identities of bacteria to starch fermentation in an in vitro model of the large intestine by RNA-based stable isotope probing.. Environ Microbiol.

[35] Leitch EC, Walker AW, Duncan SH, Holtrop G, Flint HJ (2007). Selective colonization of insoluble substrates by human faecal bacteria.. Environ Microbiol.

[36] Bird AR, Topping DL, Versalovic J, Wilson M (2008). Resistant starch as a prebiotic.. Therapeutic microbiology: Prebiotics and related strategies.

[37] Cho KH, Cho D, Wang GR, Salyers AA (2001). New regulatory gene that contributes to control of Bacteroides thetaiotaomicron starch utilization genes.. J Bacteriol.

[38] D'Elia JN, Salyers AA (1996). Contribution of a neopullulanase, a pullulanase, and an alpha-glucosidase to growth of Bacteroides thetaiotaomicron on starch.. J Bacteriol.

[39] Duncan SH, Belenguer A, Holtrop G, Johnstone AM, Flint HJ (2007). Reduced dietary intake of carbohydrates by obese subjects results in decreased concentrations of butyrate and butyrate-producing bacteria in feces.. Appl Environ Microbiol.

[40] Flint HJ, Bayer EA, Rincon MT, Lamed R, White BA (2008). Polysaccharide utilization by gut bacteria: potential for new insights from genomic analysis.. Nat Rev Microbiol.

[41] Ryan KJ, Ryan KJ, Ray CG (2004). Normal Microbial Flora.. Medical Microbiology An intorduction to infectious diseases. 4th ed.

[42] Schell MA, Karmirantzou M, Snel B, Vilanova D, Berger B (2002). The genome sequence of Bifidobacterium longum reflects its adaptation to the human gastrointestinal tract.. Proc Natl Acad Sci U S A.

[43] Xu J, Bjursell MK, Himrod J, Deng S, Carmichael LK (2003). A genomic view of the human-Bacteroides thetaiotaomicron symbiosis.. Science.

[44] Tap J, Mondot S, Levenez F, Pelletier E, Caron C (2009). Towards the human intestinal microbiota phylogenetic core.. Environ Microbiol.

[45] Crittenden R, Laitila A, Forssell P, Matto J, Saarela M (2001). Adhesion of bifidobacteria to granular starch and its implications in probiotic technologies.. Appl Environ Microbiol.

[46] Ramsay AG, Scott KP, Martin JC, Rincon MT, Flint HJ (2006). Cell-associated alpha-amylases of butyrate-producing Firmicute bacteria from the human colon.. Microbiology.

[47] McIntyre A, Gibson PR, Young GP (1993). Butyrate production from dietary fibre and protection against large bowel cancer in a rat model.. Gut.

[48] Scheppach W, Sommer H, Kirchner T, Paganelli GM, Bartram P (1992). Effect of butyrate enemas on the colonic mucosa in distal ulcerative colitis.. Gastroenterology.

[49] Ley RE, Peterson DA, Gordon JI (2006). Ecological and evolutionary forces shaping microbial diversity in the human intestine.. Cell.

[50] Turnbaugh PJ, Ley RE, Mahowald MA, Magrini V, Mardis ER (2006). An obesity-associated gut microbiome with increased capacity for energy harvest.. Nature.

[51] Cani PD, Delzenne NM (2009). Interplay between obesity and associated metabolic disorders: new insights into the gut microbiota.. Curr Opin Pharmacol.

[52] Woo KS, Seib PS (2002). Cross-linked resitant starch: Preparation and properties.. Cereal Chem.

[53] Cole JR, Wang Q, Cardenas E, Fish J, Chai B (2009). The Ribosomal Database Project: improved alignments and new tools for rRNA analysis.. Nucleic Acids Res.

[54] Edgar RC (2004). MUSCLE: multiple sequence alignment with high accuracy and high throughput.. Nucleic Acids Res.

[55] Tamura K, Dudley J, Nei M, Kumar S (2007). MEGA4: Molecular Evolutionary Genetics Analysis (MEGA) software version 4.0.. Mol Biol Evol.

[56] Hall TA (1999). BioEdit: a user-friendly biological sequence alignment editor analysis program for Windows 95/98/NT.. Nucleic Acids Symposium Series.

[57] Walter J, Hertel C, Tannock GW, Lis CM, Munro K (2001). Detection of Lactobacillus, Pediococcus, Leuconostoc, and Weissella species in human feces by using group-specific PCR primers and denaturing gradient gel electrophoresis.. Appl Environ Microbiol.

[58] Walter J, Tannock GW, Tilsala-Timisjarvi A, Rodtong S, Loach DM (2000). Detection and identification of gastrointestinal Lactobacillus species by using denaturing gradient gel electrophoresis and species-specific PCR primers.. Appl Environ Microbiol.

[59] Rinttila T, Kassinen A, Malinen E, Krogius L, Palva A (2004). Development of an extensive set of 16S rDNA-targeted primers for quantification of pathogenic and indigenous bacteria in faecal samples by real-time PCR.. J Appl Microbiol.

