# Supplementary material for: Resistant Starches Types 2 and 4 Have Differential Effects on the Composition of the Fecal Microbiota in Human Subjects
Source: PLoS One. 2010 Nov 29;5(11):e15046. doi: 10.1371/journal.pone.0015046 (PMC2993935; doi:10.1371/journal.pone.0015046)
Supplement: Table S1 — Enumeration of bacterial groups through culturing. (DOC) [file pone.0015046.s001.doc]

Table S1. Enumeration of bacterial groups through culturing.

|  | Log10 cfu/g feces (Mean ± SD) | | | | | |
| --- | --- | --- | --- | --- | --- | --- |
| Bacterial group | | RS2 | RS4 | Control | Background^a^ | P-value |
| Lactose fermenting  enterobacteria | | 6.55 ± 0.83 | 5.87 ± 1.07 | 6.77 ± 0.76 | 6.25 ± 0.51 | 0.0849 |
| Enterococci | | 4.05 ± 1.30 | 3.92 ± 1.22 | 4.64 ± 0.95 | 3.96 ± 0.41 | 0.2747 |
| Bifidobacteria | | 9.61 ± 0.50 | **9.79 ± 0.51** | *9.49 ± 0.42* | 9.57 ± 0.19 | 0.0193 |
| Bacteroides | | *9.35 ± 0.41* | **9.73 ± 0.43** | *9.38 ± 0.25* | 9.30 ± 0.33 | 0.0117 |
| Total anaerobes | | 10.51 ± 0.27 | 10.61 ± 0.33 | 10.45 ± 0.12 | 10.41 ± 0.14 | 0.2791 |

Numbers in bold represent the significantly higher treatment(s) and the ones in italic the significantly lower treatment(s).

^a^ Background refers to bacterial numbers obtained from the combined baseline and wash-out periods
