# Supplementary material for: Resistant Starches Types 2 and 4 Have Differential Effects on the Composition of the Fecal Microbiota in Human Subjects
Source: PLoS One. 2010 Nov 29;5(11):e15046. doi: 10.1371/journal.pone.0015046 (PMC2993935; doi:10.1371/journal.pone.0015046)
Supplement: Table S2 — Mean ± standard deviations of weekly symptoms reported by the subjects in a scale from 1 (best) to 5 (worse). (DOC) [file pone.0015046.s002.doc]

TableS2. Mean ± standard deviations of weekly symptoms reported by the subjects in a scale from 1 (best) to 5 (worse)

|  | RS2 | RS4 | Control | None |
| --- | --- | --- | --- | --- |
| Bowel movement | 1.73 ± 0.83 | 1.90 ± 0.93 | 1.73 ± 0.77 | 1.74 ± 0.59 |
| Stool consistency | 2.07 ± 1.29 | 2.23 ± 0.92 | 2.03 ± 0.91 | 2.00 ± 0.83 |
| Discomfort | 1.65 ± 0.65 | 1.87 ± 0.79 | 1.50 ± 0.55 | 1.53 ± 0.40 |
| Flatulence^*^ | 2.42 ± 1.28 | 2.27 ± 1.00 | 1.37 ± 0.58 | 1.54 ± 0.43 |
| Abdominal pain | 1.63 ± 0.79 | 1.47 ± 0.67 | 1.40 ± 0.60 | 1.36 ± 0.62 |
| Bloating | 1.67 ± 0.98 | 1.40 ± 0.52 | 1.07 ± 0.14 | 1.29 ± 0.45 |

^*^ Significant differences were detected by ANOVA (P < 0.05). Tukey’s post-hoc test did not detect significance in pair wise comparisons.
