# Supplementary material for: Resistant Starches Types 2 and 4 Have Differential Effects on the Composition of the Fecal Microbiota in Human Subjects
Source: PLoS One. 2010 Nov 29;5(11):e15046. doi: 10.1371/journal.pone.0015046 (PMC2993935; doi:10.1371/journal.pone.0015046)
Supplement: Table S3 — Formulation of crackers per 100 grams. (DOC) [file pone.0015046.s003.doc]

Table S3. Formulation of crackers per 100 grams

| Ingredient | Control | RS2 | RS4 |
| --- | --- | --- | --- |
| Pastry flour | 1.97 | 1.97 | 1.97 |
| Midsol 50 (native starch) | 55.72 | - | 16.39 |
| Hi-Maize 260 | - | 55.72 | - |
| Fibersym ® RW | - | - | 39.33 |
| Soybean & cottonseed shortening | 7.87 | 7.87 | 7.87 |
| Wheat gluten | 7.87 | 7.87 | 7.87 |
| White granulated sugar | 5.24 | 5.24 | 5.24 |
| Malt extract | 0.33 | 0.33 | 0.33 |
| Sweet whey-dried | 0.98 | 0.98 | 0.98 |
| Table salt | 0.66 | 0.66 | 0.66 |
| Baking soda | 0.33 | 0.33 | 0.33 |
| Water | 18.36 | 18.36 | 18.36 |
| Ammonium bicarbonate | 0.66 | 0.66 | 0.66 |
