# Supplementary material for: Resistant Starches Types 2 and 4 Have Differential Effects on the Composition of the Fecal Microbiota in Human Subjects
Source: PLoS One. 2010 Nov 29;5(11):e15046. doi: 10.1371/journal.pone.0015046 (PMC2993935; doi:10.1371/journal.pone.0015046)
Supplement: Table S4 — Baking conditions (°F) of crackers containing control starch, RS2, RS4. (DOC) [file pone.0015046.s004.doc]

Table S4. Baking conditions (°F) of crackers containing control starch, RS2, RS4

| Control Doughs Oven Profile: | | | |
| --- | --- | --- | --- |
|  | Zone 1 | Zone 2 | Zone 3 |
| Top | 460 | 470 | 420 |
| Bottom | 430 | 400 | 380 |
| Bake Time | 7 minutes |  |  |
|  |  |  |  |
| RS4 Doughs Oven Profile: | | | |
|  | Zone 1 | Zone 2 | Zone 3 |
| Top | 470 | 450 | 440 |
| Bottom | 470 | 470 | 440 |
| Bake Time | 7 minutes |  |  |
|  |  |  |  |
| RS2 Doughs Oven Profile: | | | |
|  | Zone 1 | Zone 2 | Zone 3 |
| Top | 470 | 450 | 440 |
| Bottom | 470 | 470 | 440 |
| Bake Time | 8 minutes |  |  |
