# Supplementary figures and images for: Resistant Starches Types 2 and 4 Have Differential Effects on the Composition of the Fecal Microbiota in Human Subjects
Source: PLoS One. 2010 Nov 29;5(11):e15046. doi: 10.1371/journal.pone.0015046 (PMC2993935; doi:10.1371/journal.pone.0015046)

A

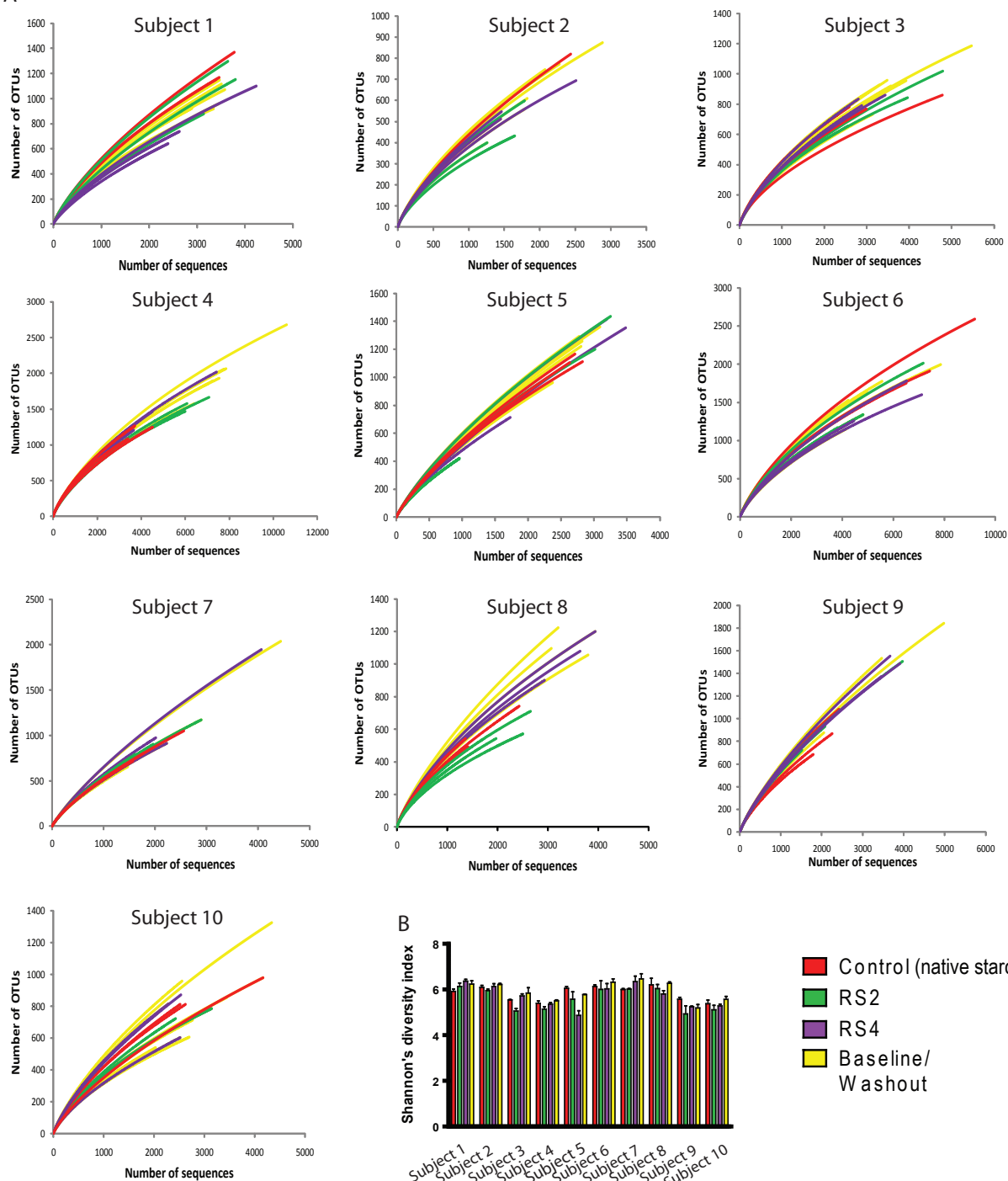

B

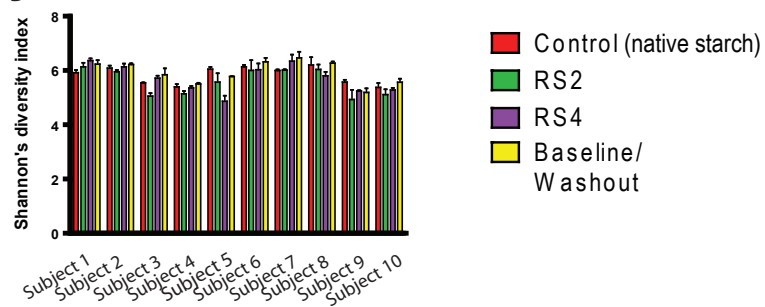

Supplement: Figure S1 — Diversity and species richness of the fecal microbiota in ten human subjects that consumed crackers containing native starch (red), RS2 (green), RS4 (purple), or no crackers (yellow). (A) Rarefaction curves showing the amount of OTUs in all individual fecal samples taken from the ten subjects. (B) Shannon's Diversity Index for all subjects during treatments and baseline/washout. (PDF) [file pone.0015046.s005.pdf]

A

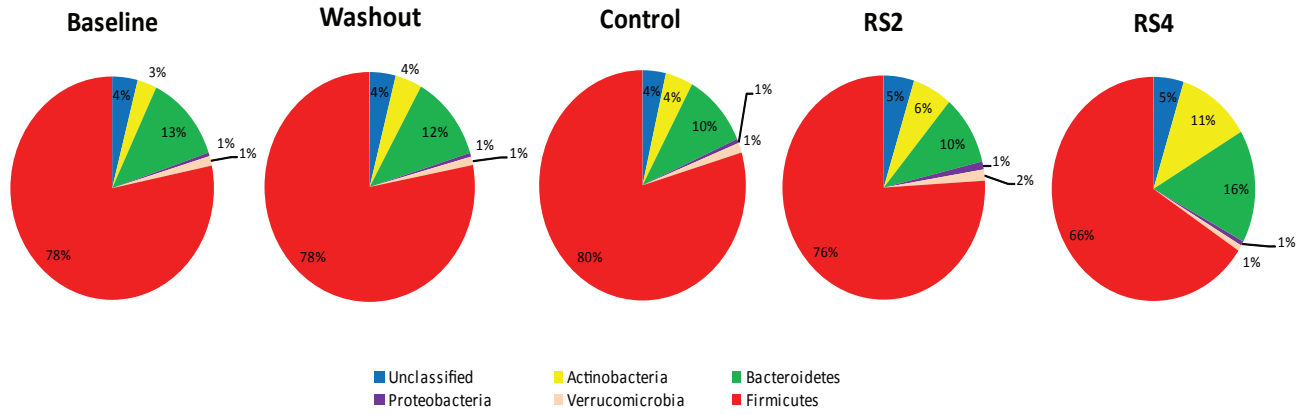

B

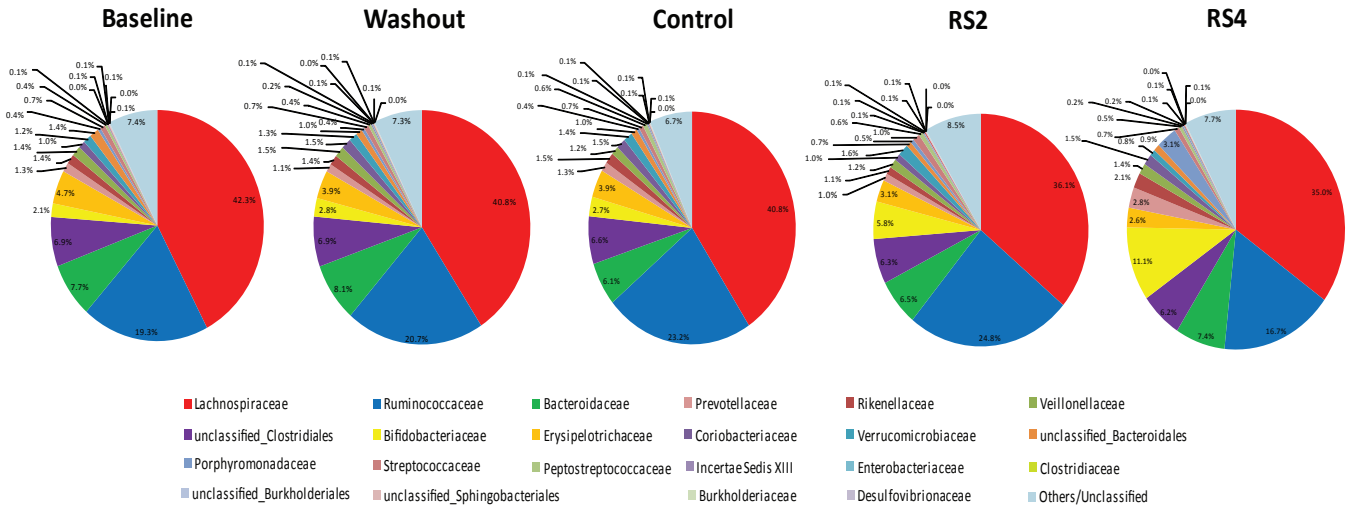

C

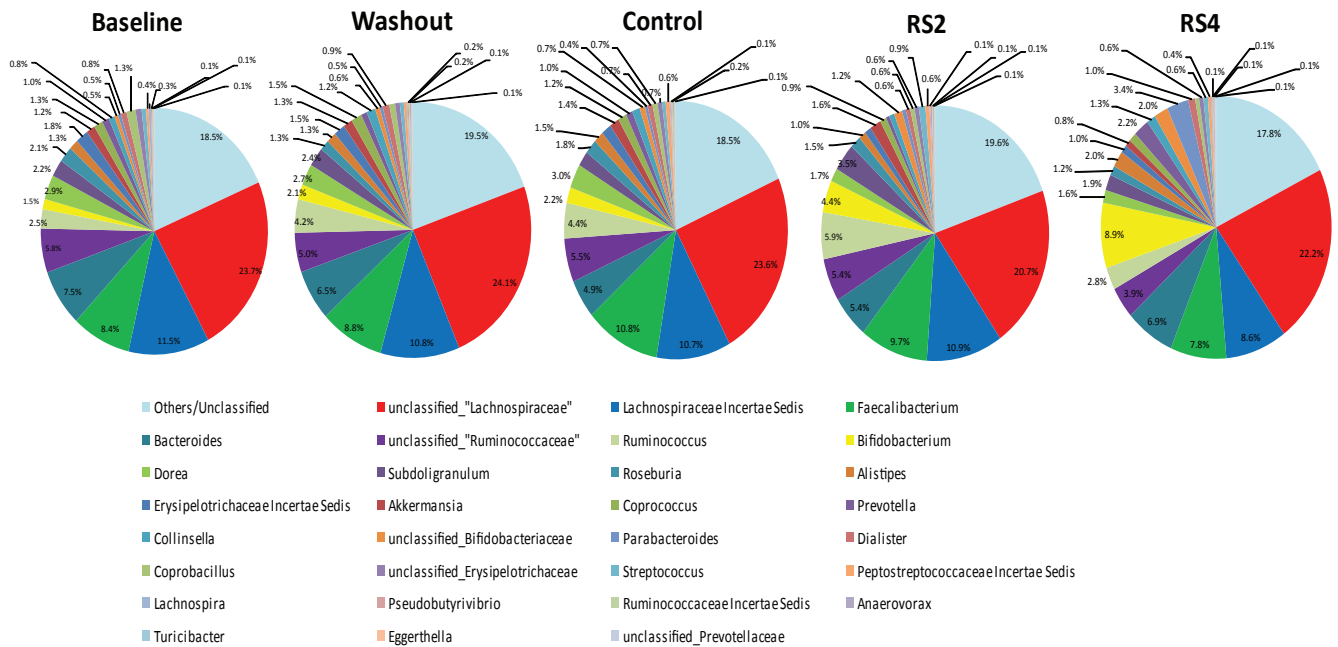

Supplement: Figure S2 — Collective fecal microbial composition including the major taxonomic groups at the (A) phylum, (B) family, and (C) genus levels averaged for 10 human subjects corresponding to the baseline, washouts, and periods in which crackers containing native starch (control), RS2 and RS4 were consumed. (PDF) [file pone.0015046.s006.pdf]

A

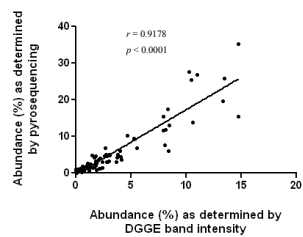

B

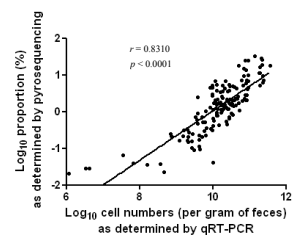

Supplement: Figure S3 — Confirmation of findings obtained with pyrosequencing by analyzing the fecal microbiota with PCR-DGGE and Bifidobacterium specific qRT-PCR. (A) Pearson correlation between the abundance of Bifidobacterium adolescentis as determined by band intensity in PCR-DGGE and pyrosequencing of 16S rRNA tags. (B) Pearson correlation between cell numbers and percent abundance of bifidobacteria as determined by qRT-PCR and pyrosequencing, respectively. (PDF) [file pone.0015046.s007.pdf]
